# Incidence and outcomes of acute respiratory distress syndrome in intensive care units of mainland China: a multicentre prospective longitudinal study

**DOI:** 10.1186/s13054-020-03112-0

**Published:** 2020-08-20

**Authors:** Xu Huang, Ruoyang Zhang, Guohui Fan, Dawei Wu, Haining Lu, Daoxin Wang, Wang Deng, Tongwen Sun, Lihua Xing, Shaohua Liu, Shilei Wang, Ying Cai, Ye Tian, Yi Zhang, Jingen Xia, Qingyuan Zhan, the CHARDSnet group, Lixin Xie, Ying Wang, Li Weng, Guangfa Zhu, Yan Liu, Man Song, Yanming Zhao, Jing Chen, Hongwen Zhao, Haijia Hou, Jingping Yang, Rina Wu, Xiyuan Xu, Xixin Yan, Haibo Xu, Dawei Wu, Haining Lu, Gengyun Sun, Dan Zhang, Beilei Zhao, Binhai Pan, Jialin Liu, Ruoming Tan, Pinhua Pan, Rongli Lu, Hong Luo, Han Zhang, Daoxin Wang, Wang Deng, Yusheng Chen, Fengfeng Lu, Sicheng Xu, Xia Luo, Hong Teng, Lijuan Chen, Lihua Xing, Shilei Wang, Tongwen Sun, Shaohua Liu, Bing Han, Yunlu Li

**Affiliations:** 1 Graduate School Chinese Academy of Medical Sciences and Peking Union Medical College, Beijing, China; 2 Department of Pulmonary and Critical Care Medicine, Center of Respiratory Medicine, China-Japan Friendship Hospital, No 2, East Yinghua Road, Chaoyang District, Beijing, China; 3 National Clinical Research Center for Respiratory Diseases, Beijing, China; 4 Institute of Respiratory Medicine, Chinese Academy of Medical Sciences, Beijing, China; 5 Department of Pulmonary Medicine, Capital Medical University, Beijing, China; 6 Institute of Clinical Medical Sciences, China-Japan Friendship Hospital, Beijing, China; 7 Department of Critical Care Medicine, Qilu Hospital of Shandong University (Qingdao), Qingdao, China; 8 Department of Respiratory and Critical Care Medicine, The Second Affiliated Hospital of Chongqing Medical University, Chongqing, China; 9 Intensive Care Unit, The First Affiliated Hospital of Zhengzhou University, Zhengzhou, China; 10 Department of Respiratory and Critical Care Medicine, The First Affiliated Hospital of Zhengzhou University, Zhengzhou, China

**Keywords:** Incidence, Acute respiratory distress syndrome (ARDS), Diagnosis, Lung protective mechanical ventilation

## Abstract

**Objectives:**

To evaluate the incidence and mortality of acute respiratory distress syndrome (ARDS) in medical/respiratory intensive care units (MICUs/RICUs) to assess ventilation management and the use of adjunct therapy in routine clinical practice for patients fulfilling the Berlin definition of ARDS in mainland China.

**Methods:**

This was a multicentre prospective longitudinal study. Patients who met the Berlin definition of ARDS were included. Baseline data and data on ventilator management and the use of adjunct therapy were collected.

**Results:**

Of the 18,793 patients admitted to participating ICUs during the study timeframe, 672 patients fulfilled the Berlin ARDS criteria and 527 patients were included in the analysis. The most common predisposing factor for ARDS in 402 (77.0) patients was pneumonia. The prevalence rates were 9.7% (51/527) for mild ARDS, 47.4% (250/527) for moderate ARDS, and 42.9% (226/527) for severe ARDS. In total, 400 (75.9%) patients were managed with invasive mechanical ventilation during their ICU stays. All ARDS patients received a tidal volume of 6.8 (5.8–7.9) mL/kg of their predicted body weight and a positive end-expository pressure (PEEP) of 8 (6–12) cmH_2_O. Recruitment manoeuvres (RMs) and prone positioning were used in 61 (15.3%) and 85 (16.1%) ventilated patients, respectively. Life-sustaining care was withdrawn from 92 (17.5%) patients. When these patients were included in the mortality analysis, 244 (46.3%) ARDS patients (16 (31.4%) with mild ARDS, 101 (40.4%) with moderate ARDS, and 127 (56.2%) with severe ARDS) died in the hospital.

**Conclusions:**

Among the 18 ICUs in mainland China, the incidence of ARDS was low. The rates of mortality and withdrawal of life-sustaining care were high. The recommended lung protective strategy was followed with a high degree of compliance, but the implementation of adjunct treatment was lacking. These findings indicate the potential for improvement in the management of patients with ARDS in China.

**Trial registration:**

Clinicaltrials.gov NCT02975908. Registered on 29 November 2016—retrospectively registered.

## Introduction

In 1967, Ashbaugh and colleagues [1] proposed a new syndrome in adults called acute respiratory distress syndrome (ARDS). Since then, many studies have investigated ARDS. In 2012, the Berlin ARDS definition [2] was published and found that the greater the severity of ARDS was, the higher the mortality rate would be. To decrease the mortality rate of ARDS, researchers have attempted to improve and implement respiratory support strategies, including incorporating a small tidal volume [3], high positive end-expiratory pressure (PEEP) [4], prone position ventilation [5], the lung recruitment manoeuvre [6], the use of neuromuscular blockers [7], high-frequency oscillatory ventilation (HFOV) [8, 9], and extracorporeal membrane oxygenation (ECMO) [10–12]. Some of the techniques have resulted in excellent progress while others were still with uncertain effect. However, there is limited information on the use of these strategies in the treatment of ARDS patients, and prospective studies from mainland China are especially lacking [13, 14].

We aimed to address some clinically important questions regarding ARDS epidemiology and management in mainland China. To date, very few studies [14] have mentioned the incidence of ARDS in some regions of China, let alone the use of lung protective interventions and adjuncts. Providing insight into the use of these interventions could enable the development of more effective interventions in clinical practice.

Therefore, we undertook the CHARDS (China Acute Respiratory Distress Syndrome epidemiology) study to assess the medical/respiratory ICU epidemiology and respiratory support of ARDS and to understand how clinicians use mechanical ventilation and adjunctive interventions in routine clinical practice.

## Methods

### Study aim, design, and setting

This was a multicentre, prospective longitudinal study. The aim of the study was to evaluate the incidence and mortality of ARDS in medical/respiratory intensive care units (MICUs/RICUs), to assess ventilation management and the use of adjunct therapy in routine clinical practice for patients fulfilling the Berlin definition of ARDS in mainland China. The study was approved by the institutional ethics committees of the participating centres. We conducted this trial in 18 ICUs in mainland China from March 2016 to February 2018. The study protocol was approved by the China-Japan Friendship Hospital ethics committee. Informed consent was obtained from all included patients. The funding source (CAMS Innovation Fund for Medical Sciences and Beijing Municipal Science and Technology Project) is an academic nonprofit organization that played no role in the study. We aimed to recruit a broadly representative sample of medical/respiratory ICUs in mainland China.

All patients met the ARDS Berlin definition for the first incidence of ARDS [2] and were admitted from March 1, 2016, to February 28, 2018, to 18 MICUs. The exclusion criteria were age younger than 18 years; chronic respiratory failure due to chronic respiratory diseases, such as chronic obstructive pulmonary disease; bronchiectasis or lung fibrosis; or inability or unwillingness to provide informed consent.

### Data collection and quality control

Day 1 was defined as the first day that the ARDS criteria were met after ICU admission. The case report form prompted investigators to provide an expanded data set for days 1, 2, 3, 4, 5, 6, 7, 8, 9, 10, 11, 12, 13, 14, 21, and 28 or at ICU discharge, hospital discharge, or death. The acute physiology and chronic health evaluation (APACHE) II score and sequential organ failure assessment (SOFA) score were recorded using data from the first 24 h in the ICU. SOFA scores ≥ 3 referring to one organ were defined as failure of that organ. Fluid balance, including daily input and output, was recorded from day 1 until day 14. All data were recorded as close as possible to 8 AM each day. Patient outcomes included the date of ICU discharge and the date of hospital discharge.

Before data entry, all the site investigators were trained to fill in the case report form. During data entry, two supervisors checked the quality of the case report forms and provided feedback to the investigators. In addition, prior to analysis, all data were screened for potentially erroneous data and outliers. These data were verified or corrected by the site investigators.

### Identification and recognition of ARDS

The diagnosis of ARDS was made by clinicians according to the Berlin ARDS definition [2] as follows: (1) the presence of acute hypoxaemic respiratory failure; (2) onset within 1 week of insult or the presence of new (within 7 days) or worsening respiratory symptoms; (3) bilateral opacities on chest X-ray or computed tomography not fully explained by effusions, lobar or lung collapse, or nodules; and (4) cardiac failure that was not the primary cause of acute hypoxaemic respiratory failure. All ICU patients were screened daily for ARDS. The patients who had acute hypoxaemia with PaO_2_/FIO_2_ (P/F) ≤ 300 mmHg were screened for ARDS. Chest X-ray or chest tomography was performed when P/F ≤ 300 mmHg (chest X-ray or chest tomography performed before the day of screening was also allowable), and the patients were managed with noninvasive or invasive ventilation with PEEP or CPAP≥ 5 cmH2O. The arterial blood gas analysis was repeated 15 min after ventilation and confirmed the P/F. The investigators then diagnosed the ARDS when the patients met the above criteria and signed consent forms, and subsequently, the investigators completed the case report forms.

### ARDS severity and mechanical ventilation parameters

Patients with ARDS undergoing noninvasive or invasive ventilation were categorized on the day of ARDS diagnosis based on their PaO_2_/FIO_2_ ratios into mild (200 < PaO_2_/FIO_2_ ≤ 300 mmHg), moderate (100 < PaO_2_/FIO_2_ ≤ 200 mmHg), and severe (PaO_2_/FIO_2_ ≤ 100 mmHg) according to the Berlin definition [2]. Moderate or severe ARDS patients who underwent noninvasive ventilation were also included in our study due to a lack of clarity in the Berlin definition. The end-inspiratory plateau pressure was measured during the first 24 h after invasive ventilation. This value was determined by application of an end-inspiratory pause of sufficient time (at least 3.0 s) to ensure airway pressure equilibrium. The investigators were encouraged to use sedatives or neuromuscular blockers to eliminate spontaneous breathing. Invasive ventilator-free days were calculated as the number of days from weaning from invasive ventilation to day 28. Patients who died before weaning were considered to have a ventilator-free-day value of 0.

### General management

The clinician decided the methods of ventilation, noninvasive positive pressure ventilation (NPPV) or invasive positive pressure ventilation (IPPV). It was recommended that all ARDS patients be ventilated with a tidal volume of 5–8 ml/kg predicted body weight, a plateau pressure less than 30 cmH_2_O, and with PEEP and FIO_2_ combinations to maintain PaO_2_ above 55 mmHg or SpO_2_ above 88% before this study.

### Outcome measures and statistical analysis

The primary goal of this study was the MICU/RICU incidence of ARDS in mainland China. Secondary outcomes included the ventilatory management of ARDS, the use of adjunctive interventions in routine clinical practice, and the ICU and hospital mortality of patients with ARDS.

Descriptive statistics included proportions for categorical variables and the mean (standard deviation) or median (interquartile range [IQR]) for continuous variables. No assumptions were made for missing data. For each parameter for which data points were missing, the value was omitted, and the denominator was adjusted accordingly. Data were unadjusted unless specifically stated otherwise. Proportions were compared using the *χ*^2^ or Fisher’s exact tests, and continuous variables were compared using the *t* test or Wilcoxon rank sum test, as appropriate. A two-sided *p* value no greater than 0.05 was considered statistically significant. Logistic regression models were used to determine the effect of prognostic factors on hospital death by means of stepwise backward elimination procedures, after adjusting for covariates of which the *p* values were less than 0.05. Statistical analyses were conducted using SAS software, version 9.4 (SAS Institute Inc.), unless otherwise indicated.

## Results

### Participating ICUs and enrolled patients

**Fig. 1:**
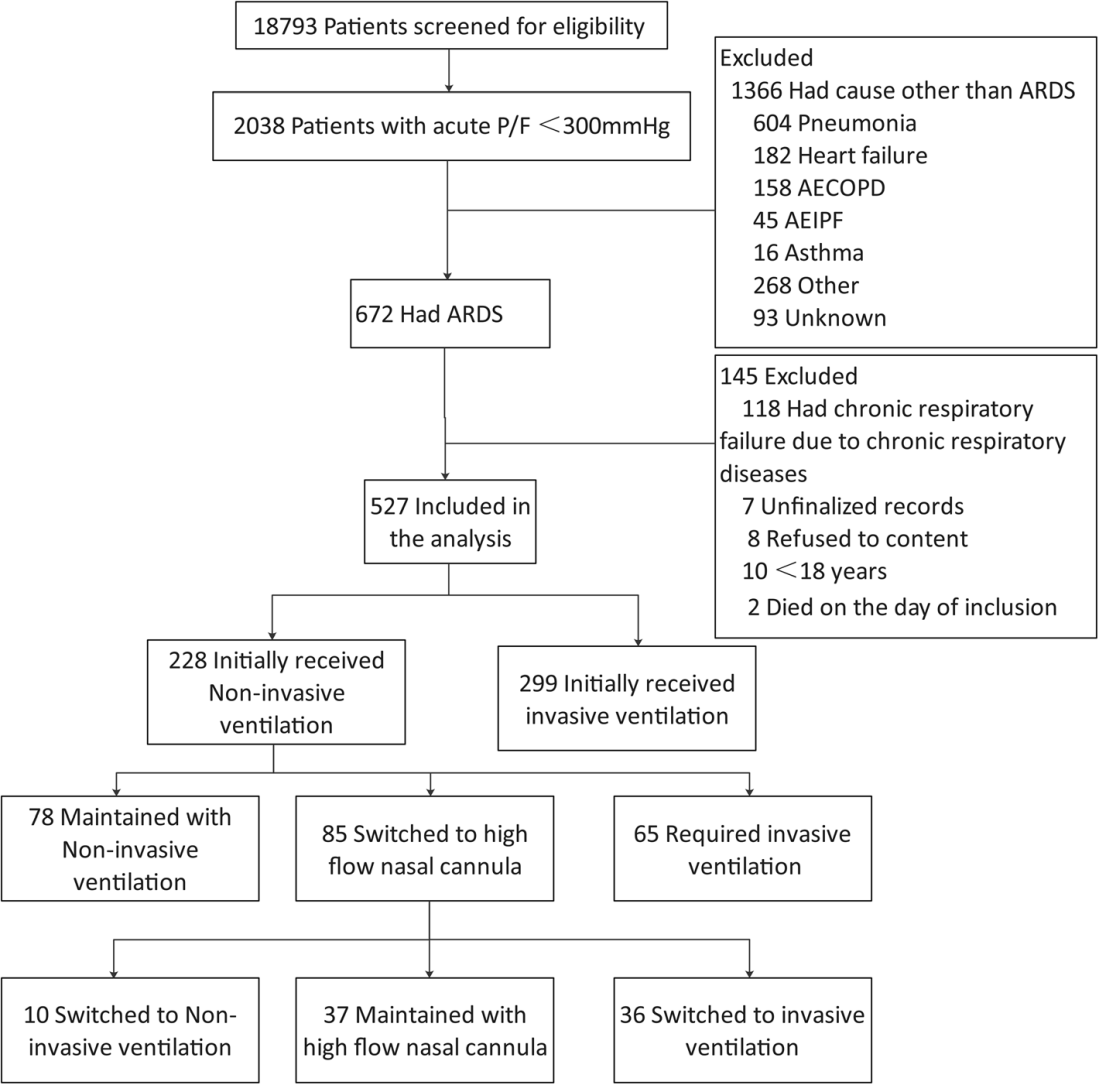
Flow of patient screening and enrollment

**Fig. 2:**
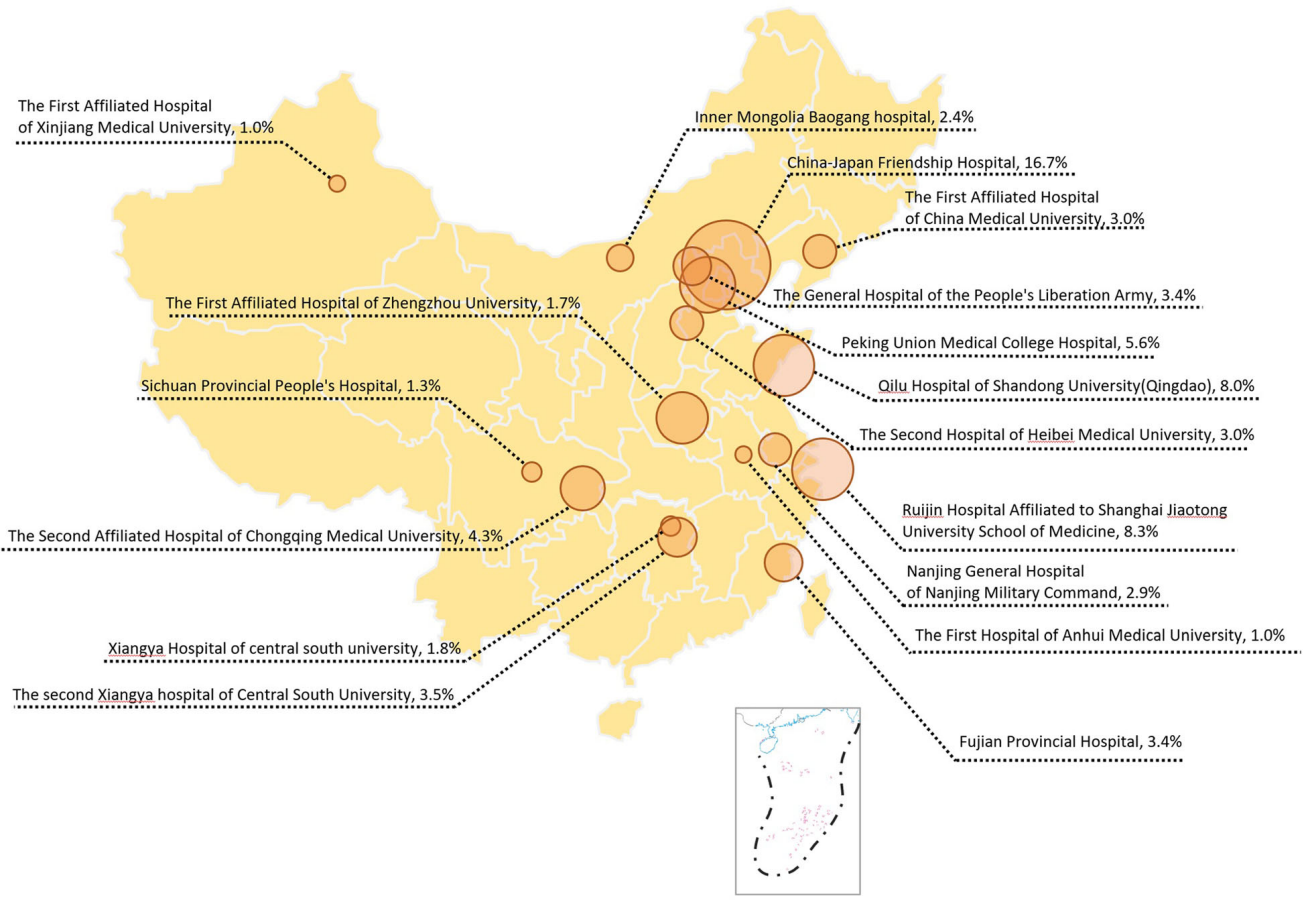
Incidences of ARDS in different ICUs. The incidences of ARDS varied among different ICUs, with the highest incidence of 16.7% in China-Japan Friendship Hospital and the lowest incidence of 1.0% in the First Affiliated Hospital of Anhui Medical University

All participating centres were closed ICUs in tertiary teaching hospitals in metropolitan cities managed by full-time ICU doctors (see eAppendix 1 and eTable 1). Eighteen ICUs from 17 hospitals in different areas of mainland China were included (see Fig. 2). Of the 18,793 patients admitted to these ICUs during the enrolment period, 672 patients were diagnosed with ARDS according to the Berlin definition and 527 were analysed (Fig. 1). Table 1 outlines their main characteristics. In total, 527 patients were included in this study. The mean age was 55.2 ± 17.4 years, and 70% were males. The mean APACHE II and SOFA scores were 17.2 ± 7.8 and 7.4 ± 3.8, respectively. Most of the patients were from the emergency room or other wards, and the medical expenses were covered by medical insurance or rural cooperative medical care (eTable 2).

**Table 1:** Characteristics of patients with acute respiratory distress syndrome

**Parameter**	**ARDS**	**Mild**	**Moderate**	**Severe**	***P*** **value**^**a**^
	***n*** **= 527**	***n*** **= 51**	***n*** **= 250**	***n*** **= 226**	
APACHEII	17.2 ± 7.8	15.8 ± 8.6	16.1 ± 7.4	18.8 ± 7.8	0.000
SOFA	7.4 ± 3.8	6.4 ± 3.8	6.8 ± 3.7	8.2 ± 3.9	0.000
Age, mean, years	55.2 ± 17.4	52.7 ± 17.8	54.2 ± 17.6	57.0 ± 17.0	0.065
Men, no. (%)	369 (70.0)	26 (51.0)	185 (74.0)	158 (69.9)	0.005
BMI	24.2 ± 4.6	23.6 ± 5.1	24.3 ± 4.6	24.2 ± 4.4	0.282
Obesity, no. (%)	65 (12.4)	4 (8.0)	29 (11.7)	32 (14.2)	0.4297
Chronic lung diseases	30 (5.7)	0 (0.0)	15 (6.0)	15 (6.7)	0.151
Hypertension	176 (33.6)	12 (23.5)	79 (31.6)	85 (38.1)	0.090
Diabetes	101 (19.3)	8 (15.7)	56 (22.4)	37 (16.7)	0.228
Coronary diseases	57 (10.9)	0 (0.0)	25 (10.0)	32 (14.3)	0.010
Chronic cardiac failure	26 (5.0)	2 (3.9)	14 (5.6)	10 (4.5)	0.806
Cerebral vascular diseases	52 (9.9)	1 (2.0)	32 (12.8)	19 (8.5)	0.040
Chronic renal failure	56 (10.7)	5 (9.8)	22 (8.8)	29 (13.1)	0.315
Liver cirrhosis	21 (4.0)	1 (2.0)	14 (5.6)	6 (2.7)	0204
Connective tissue diseases	35 (6.7)	1 (2.0)	20 (8.0)	14 (6.3)	0.275
Active neoplasm	41 (7.8)	5 (9.8)	16 (6.4)	20 (9.0)	0.500
Alcohol use disorder	21 (4.0)	2 (3.9)	15 (6.0)	4 (1.8)	0.051
Smoking	180 (34.4)	15 (29.4)	90 (36.0)	75 (33.6)	0.636

*APACHE* acute physiology and chronic health evaluation, *SOFA* sequential organ failure assessment, *BMI* body weight index

^a^*P* value represents comparisons across the ARDS severity categories for each variable

### ICU incidence of ARDS

In total, 672 fulfilled the ARDS criteria during their ICU stays. ARDS represented 3.57% of total ICU admissions, but there were large variations between different ICUs (see Fig. 2). Among the 527 ARDS patients included in the final analysis, the prevalence rates of mild, moderate, and severe ARDS were 9.7% (51/527), 47.4% (250/527), and 42.9% (226/527), respectively. 91.7 (483/527) ARDS patients were diagnosed within 24 h of ICU admission (see eFig 4). The main risk factors for ARDS were pneumonia, extrapulmonary sepsis, pancreatitis, and aspiration (77%, 7.3%, 3.4%, and 3.3%, respectively) (see Table 2). The patients’ laboratory findings are listed in eTable 3 in the supplement.

**Table 2:** Risk factors for acute respiratory distress syndrome

**Parameter**	**ARDS**	**Mild**	**Moderate**	**Severe**
	***n*** **= 527**	***n*** **= 51**	***n*** **= 250**	***n*** **= 226**
Risk factors for ARDS				
Intrapulmonary	441 (83.7)	37 (72.5)	207 (82.8)	197 (87.2)
Pneumonia	402 (77.0)	32 (64.0)	183 (73.5)	187 (83.9)
Aspiration	17 (3.3)	1 (2.0)	11 (4.4)	5 (2.2)
Drowning	3 (0.6)	0 (0.0)	3 (1.2)	0 (0.0)
Pulmonary contusion	10 (1.9)	3 (6.0)	6 (2.4)	1 (0.4)
Others	9 (1.7)	1 (2.0)	4 (1.6)	4 (1.8)
Extrapulmonary	86 (16.3)	14 (27.5)	43 (17.2)	29 (12.8)
Trauma	8 (1.5)	0 (0.0)	7 (2.8)	1 (0.4)
Extra pulmonary sepsis	38 (7.3)	7 (14.0)	18 (7.2)	13 (5.8)
Pancreatitis	18 (3.4)	5 (10.0)	9 (3.6)	4 (1.8)
Non-cardiogenic shock	1 (0.2)	0 (0.0)	1 (0.4)	0 (0.0)
Blood transfusion	2 (0.4)	1 (2.0)	1 (0.4)	0 (0.0)
Others	23 (4.4)	1 (2.0)	10 (4.0)	12 (5.4)

*ARDS* acute respiratory distress syndrome

### Mechanical ventilation management in patients with ARDS

A total of 228 ARDS patients initially received NPPV, among which 85 patients received NPPV for the diagnosis of ARDS. After diagnosis, these patients were switched to HFNC. Among the remaining 143 NPPV patients, the most popular mode was bilevel positive airway pressure (BiPAP), used for 114 (83.8%) patients, with an inspiratory positive airway pressure of 13.5 (12.0–15.0) and an expiratory positive airway pressure of 5.0 (5.0–7.0). In total, 65 of the 143 patients (45.5%) who received NPPV required invasive ventilation afterwards (see eTable 4).

In total, 299 (56.7%) ARDS patients initially received IPPV. Ventilator management varied with ARDS severity, and the ventilation modes on the first day of ARDS are shown in Table 3. Pressure control was the most selected mode, used for 192 (48%) patients. The ARDS patients who received IPPV had a median tidal volume (VT) of 6.8 (5.8–7.9) ml/kg of predicted body weight and a median PEEP of 8 (6–12) cmH_2_O on the first day of IPPV. A total of 78.25% (313/400) of patients received a tidal volume of ≤ 8 ml/kg of PBW. PEEP was relatively low overall but became progressively higher in more severe patients. The plateau pressure and driving pressure on the first day of IPPV was 20 (16–26) cmH_2_O and 12 (8–16) cmH_2_O, respectively. Finally, 400 (75.9%) of all ARDS patients required invasive mechanical ventilation, which accounted for 18.5% (400/2168) of all ventilated patients in the same period.

**Table 3:** Characteristics of ARDS patients treated with invasive ventilation and use of adjunctive by severity category

**Parameter**	**ARDS**	**Mild**	**Moderate**	**Severe**	***P*** **value**^**b**^
	***n*** **= 527**	***n*** **= 51**	***n*** **= 250**	***n*** **= 226**	
D1 IPPV	299 (56.7)	25 (49.0)	125 (50.0)	149 (65.9)	0.001
IPPV during ICU	400 (75.9)	30 (58.8)	177 (70.8)	193 (85.4)	0.000
Mode on 1st day of IPPV					
Volume control ventilation	55 (13.8)	3 (10.0)	22 (12.4)	30 (13.8)	
Pressure control ventilation	192 (48.0)	11 (36.7)	81 (45.8)	100 (51.8)	
SIMV+PS	47 (11.8)	4 (13.3)	18 (10.2)	25 (13.0)	
Pressure support ventilation	70 (17.5)	7 (23.3)	36 (20.3)	27 (14.0)	
Bilevel	32 (8.0)	5 (16.7)	18 (10.2)	9 (4.7)	
Other modes	4 (1.0)	0 (0.0)	2 (1.1)	2 (1.0)	
PEEP median (IQR), cmH_2_O	8 (6–12)	7 (5–8)	8 (6–10)	10 (6–12)	0.000
*V*_T_, median (IQR) (ml/kg PBW)	6.8 (5.8–7.9)	7.0 (6.6–7.7)	6.8 (5.9–8.0)	6.8 (5.8–7.9)	0.538
Plateau pressure, median (IQR), cmH_2_O	20 (16–26)	20 (15–23)	20 (15–25)	22 (18–27)	0.220
Driving pressure^a^, median (IQR), cmH_2_O	12 (8–16)	14 (10–15)	13 (8–16)	12 (8–17)	0.779
Airway resistance^a^, median (IQR) cmH_2_O/L/S	12.0 (8.0–18.2)	12.0 (9.7–17.0)	11.0 (7.8–19.0)	12.0 (8.0–18.0)	0.571
Compliance^a^, median (IQR) ml/cmH_2_O	35.0 (25.0–43.7)	36.4 (30.7–43.0)	36.4 (24.0–52.0)	32.0 (25.0–42.0)	0.191
ABG, D1 ARDS					
PaO_2_/FIO_2_, median (IQR), mmHg	113 (80–161)	227 (206–270)	142 (115–166)	78 (59–96)	0.000
PaCO_2_, median (IQR), mmHg	36.3 (31.2–42.7)	36.2 (29.6–39.0)	35.9 (31.0–41.5)	37.2 (31.8–45.2)	0.044
pH, median (IQR)	7.42 (7.36–7.46)	7.43 (7.36–7.48)	7.2 (7.36–7.46)	7.41 (7.40–7.46)	0.651
NMBAs	107 (26.8)	5 (16.7)	43 (24.3)	59 (30.6)	0.177
RM	61 (15.3)	2 (6.7)	26 (14.7)	33 (17.1)	0.322
PPV	85 (21.3)	3 (10.0)	29 (16.4)	53 (27.5)	0.011
ECMO	61 (15.3)	2 (6.7)	23 (13.0)	36 (18.7)	0.142
HFOV	3 (0.8)	0 (0.0)	1 (0.6)	2 (1.0)	1.000
High-dose corticosteroid^c^	157 (29.8)	13 (25.5)	59 (23.6)	85 (37.6)	0.003

*IPPV* invasive positive pressure ventilation, *ICU* intensive care unit, *SIMV* synchronized intermittent mandatory ventilation, *PS* pressure support, *PEEP* positive end-expiratory pressure, *VT* tidal volume, *PBW* predicted body weight, *ABG* arterial blood gas, *NMBAs* neuromuscular blockade, *RM* lung recruitment manoeuvre, *PPV* prone position ventilation, *EMCO* extracorporeal membrane oxygenation, *HFOV* high-frequency oscillatory ventilation

^a^Plateau pressure values, driving pressure values, airway resistance values, and respiratory compliance values are limited to patients in whom this value was reported. The number of measured patients is 211 cases. Patients receiving HFOV or ECMO were also excluded

^b^*P* value represents comparisons across the ARDS severity categories for each variable

^c^High-dose corticosteroids was defined as doses that were equal to or greater than the equivalent of 1 mg/kg of prednisolone

### Use of adjunctive measures

The use of adjunctive treatments in patients with ARDS is showed in Table 3. Neuromuscular blockade was used in 107/400 (26.8%) ventilated patients, and recruitment manoeuvres (RMs) were used in 61/400 (15.3%) ventilated patients. Prone position was used in 85/400 (21.3%) ventilated patients and increased with the severity of ARDS, in 343 ventilated patients whose PFR ≤ 150 mmHg within 1 week of ARDS, only 24.8% (85) used prone position. None of the patients received nitric oxide as an adjunctive treatment. Three of the participating centres used extracorporeal membrane oxygenation. HFOV and ECMO were used in 3/400 (0.8%) and 61/400 (15.3%) invasively ventilated ARDS patients, respectively. High-dose steroids (which were defined as doses that were equal to or greater than the equivalent of 1 mg/kg of prednisolone) were used in 160 (30.4%) ARDS patients. There was a trend in which the higher the ARDS severity was, the more positive the fluid balance would be, especially for the first 4 days after ARDS diagnosis (see eTable 5 in the supplement).

### ARDS outcomes

**Fig. 3:**
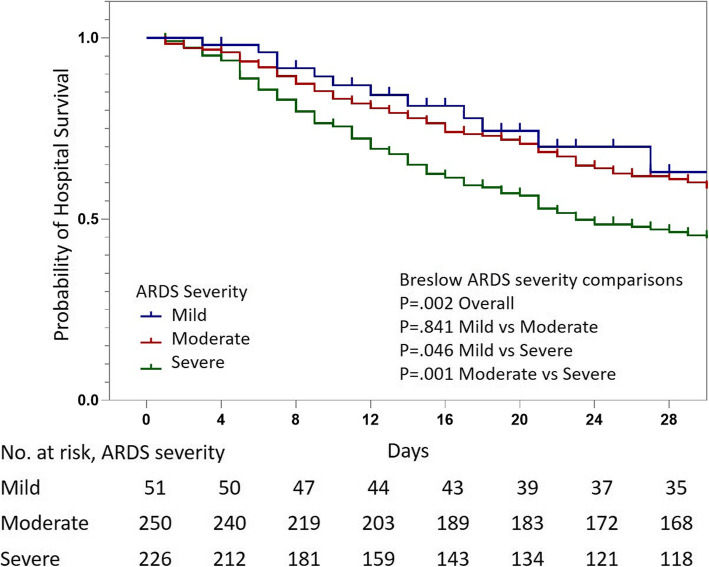
Probability of hospital survival by ARDS severity. Survival curve showed a lower likelihood of survival in severe group compared with mild and moderate groups on day 1

The severity of ARDS worsened in 154/301 (51.2%) patients with mild or moderate ARDS (Table 4). Overall, the unadjusted numbers of ICU and hospital deaths from ARDS were 232/527 (44%) and 244/527 (46.3%), respectively. The hospital mortality in mild, moderate, and severe group were 31.4%, 40.4%, and 56.2%, respectively (see Table 4). Survival curve showed a lower likelihood of survival in severe group compared with mild and moderate groups on day 1 (see Fig. 3). In total, life-sustaining care was withdrawn from 92/527 (17.5%) patients, and all of these patients died soon after withdrawal. The incidence of IPPV barotrauma was 7.8% (31/400), among which pneumothorax occurred in 23(4.3%) patients. Patients with a driving pressure of more than 15 cmH_2_O on the first day of IPPV had worse outcomes, but this was not the case for plateau pressure (eFigures 2 and 3). Shock occurred in 191 (36.3%) patients. Hospital-acquired infections occurred in 135/527 (25.6%) patients, and most infections were hospital-acquired pneumonia (117/527, 22.3%). Extrapulmonary organ failure occurred in 241/527 (45.7%) ARDS patients. The univariate analysis of survival is showed in eTable 6. The multivariate model indicated that age [hazard ratio (HR) 1.680; 95% CI 1.106–2.551; *p* = 0.015], corticosteroid within 1 month [HR 1.749, 95% CI 1.089–2.808, *p* = 0.021], driving pressure > 15 cmH_2_O [HR 1.897, 95% CI 1.210–2.974, *p* = 0.005], and shock [HR 2.017, 95% CI 1.308–3.111, *p* = 0.002] were independently significantly associated with hospital mortality.

**Table 4:** Outcomes of invasively ventilated patients by acute respiratory distress syndrome severity at diagnosis

**Parameter**	**ARDS**	**Mild**	**Moderate**	**Severe**	***P*** **value**^**a**^
	***n*** **= 527**	***n*** **= 51**	***n*** **= 250**	***n*** **= 226**	
Progression of ARDS severity					
Progression to moderate	26/51 (51.0)	26/51 (51.0)	–	–	
Progression to severe	128/301 (42.5)	16/51 (31.4)	112/250 (44.8)	–	
Invasive ventilation-free days to day 28, median (IQR), days	6 (0–22)	21 (0–28)	12 (0–23)	0 (0–18)	0.000
IPPV barotrauma	31 (7.8)	1 (3.3)	15 (8.5)	15 (7.7)	0.5621
Subcutaneous emphysema	14 (2.6)	0 (0.0)	7 (2.8)	7 (3.0)	0.2362
Mediastinal emphysema	19 (3.6)	0 (0.0)	8 (3.2)	11 (4.8)	0.0946
Pneumothorax	23 (4.3)	1 (2.0)	11 (4.4)	11 (4.8)	0.6149
HAI	135 (25.6)	9 (17.6)	58 (23.2)	68 (30.1)	0.089
HAP	117 (22.3)	6 (12.0)	50 (20.0)	61 (27.2)	0.031
CRBSI	23 (4.4)	2 (4.0)	9 (3.6)	12 (5.4)	0.640
BSI	31 (5.9)	3 (5.9)	12 (4.8)	16 (7.1)	0.5889
Intra-abdominal infection	10 (1.9)	1 (2.0)	5 (2.0)	4 (1.8)	0.983
Other HAI	15 (3.0)	1 (2.0)	8 (3.3)	6 (2.8)	0.879
Organ failure					
At least one extrapulmonary organ failure	241 (45.7)	15 (29.4)	104 (41.6)	122 (54.0)	0.001
Shock	191 (36.3)	14 (27.5)	84 (33.2)	94 (41.8)	0.058
Kidney	130 (24.7)	5 (9.8)	61 (24.4)	64 (28.4)	0.020
CRRT	119 (22.6)	10 (19.6)	54 (21.6)	55 (24.3)	0.568
Liver	57 (10.8)	5 (9.8)	22 (8.8)	30 (13.3)	0.275
Coagulation	64 (12.2)	5 (9.8)	31 (12.4)	28 (12.4)	0.863
GCS	38 (7.3)	4 (7.8)	14 (5.6)	20 (9.0)	0.378
ICU length of stay, median (IQR), days	11 (7, 21)	7 (5, 17)	12 (7, 20)	12 (6, 21)	0.080
ICU mortality^c^	232 (44.0)	15 (29.4)	96 (38.4)	121 (53.5)	0.000
Hospital length of stay, median (IQR), days	19 (10, 29)	15 (9, 23)	20 (12, 30)	17 (9, 29)	0.049
Hospital mortality^c^	244 (46.3)	16 (31.4)	101 (40.4)	127 (56.2)	0.000
Withdrawal of life sustaining care^b^	92 (17.5)	5 (9.8)	38 (15.2)	49 (21.7)	0.021
Patients except withdrawal					
ICU mortality	140 (32.2)	10 (21.7)	58 (27.4)	72 (40.7)	0.005
Hospital mortality	152 (34.9)	11 (23.9)	63 (29.7)	78 (44.1)	0.003

*ARDS* acute respiratory distress syndrome, *IPPV* invasive positive pressure ventilation, *HAI* hospital-acquired infection, *HAP* hospital-acquired pneumonia, *CRBSI* catheter-related blood stream infection, *BSI* blood stream infection, *CRRT* continuous renal replacement therapy, *GCS* Glasgow coma scale, *ICU* intensive care unit; organ failure: SOFA scores ≥ 3 referring to one organ were defined as failure of that organ

^a^*P* value represents comparisons across the ARDS severity categories for each variable

^b^All the withdrawal of life sustaining care patients discharged from the hospital were confirmed dead on the day of withdrawal

^c^When ICU and hospital mortality were calculated, the withdrawal patients were included

## Discussion

In this prospective registry study carried out in 18 ICUs in mainland China, ARDS was still an important public health problem, with a low ICU incidence, geographic variation, and high hospital mortality. The low use of recommended adjuncts and the high ratio of withdrawal of life-sustaining care, among moderate to severe ARDS patients, were also found.

In this study, the average ARDS incidence was low in the MICUs/RICUs of mainland China, and there was nearly 17-fold variation in the incidences of different ICUs. Prior epidemiological studies reported an ARDS incidence ranging from 2 to 25% of ICU patients [15–18]. In the LUNGSAFE study [15], which included ICUs from 50 countries, the geographic variation was also high, and the incidence in Asia was lower. In contrast to other studies, our study showed a lower ratio of mild ARDS and a higher ratio of moderate ARDS relative to severe ARDS, while in the LUNGSAFE study [15], the proportions of mild, moderate, and severe ARDS were 30%, 46.6%, and 23.4%, respectively. The data included in the Berlin definition [19] also showed proportions of 22%, 64%, and 14% for mild, moderate, and severe ARDS, respectively. The reason for the relatively low incidence of ARDS and high variation incidence between different ICUs in this study is threefold. First, the patients admitted to the different ICUs had different kinds of diseases. The leading ICU received severe cases of pneumonia from all over the country and had the highest incidence of ARDS, while patients in the ICUs with lower incidences of ARDS were limited to patients with chronic respiratory diseases such as COPD or interstitial lung diseases. Second, in the LUNGSAFE study [15], Bellani et al. found that ARDS was underdiagnosed, and clinician recognition of ARDS was the lowest in patients with mild ARDS (51.3%; 95% CI, 47.5–55.0%). As the severity of ARDS increased, the proportion of clinician recognition increased accordingly. In our study, some of the patients with mild ARDS may not be recognized by clinicians in general wards or emergency room and had no chance to be transferred to an ICU, which may be both the reason of low incidence of ARDS and low ratio of mild ARDS cases. Third, there were also other ICUs in most of the included hospitals, such as surgical, general, or emergency ICUs, and some of the ARDS patients (especially for extrapulmonary ARDS patients) have been admitted there. There was also the possibility that because the screening protocol was implemented by the investigators but not the computer algorithm, some ARDS cases may have been missed by investigators. Other explanations of the low incidence may be the use of different definitions of ARDS in other studies (AECC or Berlin definition) and the prospective or retrospective designs of the studies.

Since 2000, variable ventilation strategies have been proven to be effective according to ARDS mechanical ventilation guidelines, including small tidal volume, appropriate PEEP, limit plateau pressure, and prone position ventilation. Some other strategies are inconclusive and still need more investigation, including the use of neuromuscular blockers, RMs, and ECMO. In our study, these proven or recommended approaches to mechanical ventilation and adjunctive measures appeared to be underused. The implementation of low tidal volume ventilation was in the highest agreement with the guidelines. 78.25% of the patients in this study had a tidal volume of ≤ 8 ml/kg PBW recommended as the guideline, which indicates the wide adoption of implementing a small tidal volume strategy by Chinese doctors. The other two important parameters consistent with the low tidal volume are the limitation of plateau pressure and driving pressure, which guarantee the avoidance of barotrauma. We found from the multivariate regression analysis that patients with a driving pressure > 15 cmH_2_O had worse outcomes, while this was not the case for plateau pressure. This was in accordance with Amato’s study [20], which motivates us to focus more on the limitation of driving pressure. Higher PEEP was used in patients with severe ARDS compared with those with mild or moderate ARDS, as reported in prior studies [15], but concerns have been raised by the relatively low PEEP found in our study compared to the recommended PEEP in ARDSnet [3] and other studies [6]. Adjunctive measures were used infrequently for RM, prone positioning, and neuromuscular blockade. Among 343 ventilated patients with P/F < 150 mmHg within 1 week, only 85 (24.8%) patients used PPV. However, recent studies [21–23] about RM have shown only improvement for oxygenation but no benefit for mortality. It is possible that the relatively low use of adjunctive measures reflects the uncertain effect of the treatment or the low compliance to the guidelines among clinicians. Additionally, expensive measurements such as ECMO were not widely applied in the included centres (only in 3 ICUs).

Our study showed high ICU and hospital mortality rates of patients with ARDS. In fact, the mortality rates of ARDS have remained at approximately 36–50% since the syndrome was first described [6, 15, 17, 24–28]. The reason for the high mortality rate may be fourfold. First, there was a high incidence of withdrawal of life-sustaining care, which has seldom been reported before. When we excluded withdrawal patients, the hospital mortality rate was 34.9% (152/435). When we included withdrawal patients who died soon after withdrawal, the mortality rate was 46.3% (244/527). Suchyta et al. [29] found that withdrawal was more likely for patients older than 55 years (21/51) than for those 55 years or younger (3/32; *p* < 0.001). However, most of the families made such decisions due to economic reasons or based on Chinese traditions in our study. Second, there was relatively low compliance with some of the recommended guidelines. Third, the therapeutic levels varied in different regions of China. Finally, limitations in the facilities resulted in the loss of opportunities to receive further treatment, such as ECMO. We also found the hospital mortality was not different between mild and moderate group; the main reason may be that 42 in 51 mild ARDS patients progressed to moderate or severe group, and also the number of the included ARDS cases was too small to be statistically significant.

This study had a number of limitations. Although the included hospitals are general hospitals across Mainland China, most of the included ICUs are respiratory ICUs, and the risk factors may be constrained to intrapulmonary elements; therefore, the extra pulmonary element-induced ARDS patients may have been underrepresented. Additionally, the number of included ICUs was relatively small, which may have led to selection bias and could not represent the reality in mainland China, limiting the generalizability of our findings. As an epidemiological study, we could not obtain patient data from the enrolled ICUs, so it is possible that we missed some patients who met the inclusion criteria for ARDS in participating centres, especially for patients with mild ARDS. To ensure our data quality, we assigned two doctors to check the quality of the case report forms from different centres every 3 months. To standardize the inclusion procedure, we offered all the investigators web-based training and midterm conferences. Two important respiratory mechanical parameters, namely, plateau pressure and driving pressure, were reported in only 52.8% (211/400) of all IPPV patients with ARDS. The reason for so few patients undergoing this measure may be that most of them were ventilated with the spontaneous ventilation mode. Additionally, doctors did not recognize that plateau pressure and driving pressure were important parameters for ARDS patients and that refractory tachypnoea may also affect the measurement of plateau pressure and driving pressure.

## Conclusions

Among included ICUs in Mainland China, the ARDS incidence was lower than those found in other studies. Most concerning is the high mortality and withdrawal rates of life sustaining care in patients with moderate to severe ARDS. The ARDS patients were undertreated and had a relatively low level of compliance with the guidelines. The findings indicate the potential for improvement in early recognition of mild ARDS outside the ICU and standardization of ventilation management of ARDS patients.

## Supplementary information

**Additional file 1: eFig 1.** Probability of hospital survival by driving pressure. Patients with a driving pressure greater than 15 cmH_2_O on day 1 of mechanical ventilation after ARDS diagnosis had a higher mortality.

**Additional file 2: eFig 2.** Probability of hospital survival by plateau pressure. Patients with a plateau pressure of greater than 30 cmH_2_O on day 1 of mechanical ventilation after ARDS diagnosis had mortality similar to that of patients with a plateau pressure of less than 30 cmH_2_O.

**Additional file 3: eFig 3.** Logistic regression of hospital mortality.

**Additional file 4: eFig 4.** Time-to-event analysis of the time course of ARDS onset.

**Additional file 5.** Characteristics of chinese hospitals and ICUs including process of care and ICU delivery systems.

**Additional file 6: eTable 1.** Characteristics of participating centers.

**Additional file 7: eTable 2.** Resources and types of medical expenses of acute respiratory distress syndrome patients.

**Additional file 8: eTable 3.** Laboratory findings of acute respiratory distress syndrome patients.

**Additional file 9: eTable 4.** Characteristics of ARDS patients treated with noninvasive ventilation by severity category at diagnosis.

**Additional file 10: eTable 5.** Fluid balance of acute respiratory distress syndrome patients.

**Additional file 11: eTable 6.** Comparison of survivors versus non-survivors in patients with ARDS.

## Data Availability

The data that support the findings of this study are available from Qingyuan Zhan, but restrictions apply to the availability of these data, which were used under license for the current study, so these data are not publicly available. Data are, however, available from the authors upon reasonable request and with permission from the CHARDSnet group.

## Abbreviations

ARDS: Acute respiratory distress syndrome
PEEP: High positive end-expiratory pressure
HFOV: High-frequency oscillatory ventilation
ECMO: Extracorporeal membrane oxygenation
MICUs/RICUs: Medical/respiratory intensive care units
APACHE: The acute physiology and chronic health evaluation
SOFA: Sequential organ failure assessment
NPPV: Non-invasive positive pressure ventilation
IPPV: Invasive positive pressure ventilation

## References

[1] Ashbaugh DG, Bigelow DB, Petty TL, Levine BE (1967). Acute respiratory distress in adults. Lancet (London).

[2] Force ADT, Ranieri VM, Rubenfeld GD, Thompson BT, Ferguson ND, Caldwell E, Slutsky AS (2012). Acute respiratory distress syndrome: the Berlin definition. JAMA.

[3] Brower RG, Matthay MA, Morris A, Schoenfeld D, Thompson BT, Wheeler A, Acute Respiratory Distress Syndrome N (2000). Ventilation with lower tidal volumes as compared with traditional tidal volumes for acute lung injury and the acute respiratory distress syndrome. N Engl J Med.

[4] Brower RG, Lanken PN, MacIntyre N, Matthay MA, Morris A, Ancukiewicz M, Blood Institute ACTN (2004). Higher versus lower positive end-expiratory pressures in patients with the acute respiratory distress syndrome. N Engl J Med.

[5] Guerin C, Reignier J, Richard JC, Beuret P, Gacouin A, Boulain T, Ayzac L (2013). Prone positioning in severe acute respiratory distress syndrome. N Engl J Med.

[6] Meade MO, Cook DJ, Guyatt GH, Slutsky AS, Arabi YM, Cooper DJ,Lung Open Ventilation Study I: Ventilation strategy using low tidal volumes, recruitment maneuvers, and high positive end-expiratory pressure for acute lung injury and acute respiratory distress syndrome: a randomized controlled trial. JAMA 2008, 299(6):637–645. 10.1001/jama.299.6.637 18270352

[7] Papazian L, Forel JM, Gacouin A, Penot-Ragon C, Perrin G, Loundou A, Investigators AS (2010). Neuromuscular blockers in early acute respiratory distress syndrome. N Engl J Med.

[8] Young NH, Andrews PJD (2011). High-frequency oscillation as a rescue strategy for brain-injured adult patients with acute lung injury and acute respiratory distress syndrome. Neurocritical care vol 15.

[9] Ferguson ND, Cook DJ, Guyatt GH, Mehta S, Hand L, Austin P, Meade MO (2013). High-frequency oscillation in early acute respiratory distress syndrome. N Engl J Med.

[10] Zampieri FG, Mendes PV, Ranzani OT, Taniguchi LU, Pontes Azevedo LC, Vieira Costa EL, Park M (2013). Extracorporeal membrane oxygenation for severe respiratory failure in adult patients: a systematic review and meta-analysis of current evidence. J Crit Care.

[11] Peek GJ, Clemens F, Elbourne D, Firmin R, Hardy P, Hibbert C, Wilson A (2006). CESAR: conventional ventilatory support vs extracorporeal membrane oxygenation for severe adult respiratory failure. BMC health services research. vol. 6.

[12] Combes A (2011). Extracorporeal membrane oxygenation (ECMO) for severe acute respiratory distress syndrome (ARDS). The EOLIA (ECMO to rescue Lung Injury in severe ARDS) trial: A multicenter, international, randomized, controlled open trial. [French]. Reanimation. vol. 20.

[13] Liu L, Yang Y, Gao Z, Li M, Mu X, Ma X, Qiu H (2018). Practice of diagnosis and management of acute respiratory distress syndrome in mainland China: a cross-sectional study. J Thorac Dis.

[14] Lu Y, Song Z, Zhou X, Huang S, Zhu D, Yang CBX, Shanghai ASG (2004). A 12-month clinical survey of incidence and outcome of acute respiratory distress syndrome in Shanghai intensive care units. Intensive Care Med.

[15] Bellani G, Laffey JG, Pham T, Fan E, Brochard L, Esteban A, Group ET (2016). Epidemiology, patterns of care, and mortality for patients with acute respiratory distress syndrome in intensive care units in 50 countries. JAMA.

[16] Brun-Buisson C, Minelli C, Bertolini G, Brazzi L, Pimentel J, Lewandowski K, Group AS (2004). Epidemiology and outcome of acute lung injury in European intensive care units. Results from the ALIVE study. Intensive Care Med.

[17] Villar J, Blanco J, Anon JM, Santos-Bouza A, Blanch L, Ambros A, Network A (2011). The ALIEN study: incidence and outcome of acute respiratory distress syndrome in the era of lung protective ventilation. Intensive Care Med.

[18] Bersten AD, Edibam C, Hunt T, Moran J, Australian, New Zealand Intensive Care Society Clinical Trials G: Incidence and mortality of acute lung injury and the acute respiratory distress syndrome in three Australian states. Am J Respir Crit Care Med 2002, 165(4):443–448. 10.1164/ajrccm.165.4.2101124 11850334

[19] Ferguson ND, Fan E, Camporota L, Antonelli M, Anzueto A, Beale R, Ranieri VM (2012). The Berlin definition of ARDS: an expanded rationale, justification, and supplementary material. Intensive Care Med.

[20] Amato MB, Meade MO, Slutsky AS, Brochard L, Costa EL, Schoenfeld DA, Brower RG (2015). Driving pressure and survival in the acute respiratory distress syndrome. N Engl J Med.

[21] Bhattacharjee S, Soni KD, Maitra S (2018). Recruitment maneuver does not provide any mortality benefit over lung protective strategy ventilation in adult patients with acute respiratory distress syndrome: a meta-analysis and systematic review of the randomized controlled trials. J Intensive Care.

[22] Kang H, Yang H, Tong Z (2019). Recruitment manoeuvres for adults with acute respiratory distress syndrome receiving mechanical ventilation: a systematic review and meta-analysis. J Crit Care.

[23] Cavalcanti AB, Suzumura EA, Laranjeira LN, Paisani DM, Damiani LP, Ribeiro de Carvalho CR, Writing Group for the Alveolar Recruitment for Acute Respiratory Distress Syndrome Trial I (2017). Effect of lung recruitment and titrated positive end-expiratory pressure (PEEP) vs low PEEP on mortality in patients with acute respiratory distress syndrome: a randomized clinical trial. JAMA.

[24] Monchi M, Bellenfant F, Cariou A, Joly LM, Thebert D, Laurent I, Brunet F (1998). Early predictive factors of survival in the acute respiratory distress syndrome. A multivariate analysis. Am J Respir Crit Care Med.

[25] Roupie E, Lepage E, Wysocki M, Fagon JY, Chastre J, Dreyfuss D, Brochard L (1999). Prevalence, etiologies and outcome of the acute respiratory distress syndrome among hypoxemic ventilated patients. SRLF Collaborative Group on Mechanical Ventilation. Societe de Reanimation de Langue Francaise. Intensive Care Med.

[26] Esteban A, Anzueto A, Frutos F, Alia I, Brochard L, Stewart TE, Mechanical Ventilation International Study G: Characteristics and outcomes in adult patients receiving mechanical ventilation: a 28-day international study. JAMA 2002, 287(3):345–355. 10.1001/jama.287.3.345 11790214

[27] Sigurdsson MI, Sigvaldason K, Gunnarsson TS, Moller A, Sigurdsson GH (2013). Acute respiratory distress syndrome: nationwide changes in incidence, treatment and mortality over 23 years. Acta Anaesthesiol Scand.

[28] Linko R, Okkonen M, Pettila V, Perttila J, Parviainen I, Ruokonen E, group FI-s: Acute respiratory failure in intensive care units. FINNALI: a prospective cohort study. Intensive Care Med 2009, 35(8):1352–1361. 10.1007/s00134-009-1519-z 19526218

[29] Suchyta MR, Clemmer TP, Elliott CG, Orme JF, Morris AH, Jacobson J, Menlove R (1997). Increased mortality of older patients with acute respiratory distress syndrome. Chest.

