# Supplementary figures and images for: Incidence and outcomes of acute respiratory distress syndrome in intensive care units of mainland China: a multicentre prospective longitudinal study
Source: Crit Care. 2020 Aug 20;24:515. doi: 10.1186/s13054-020-03112-0 (PMC7439799; doi:10.1186/s13054-020-03112-0)

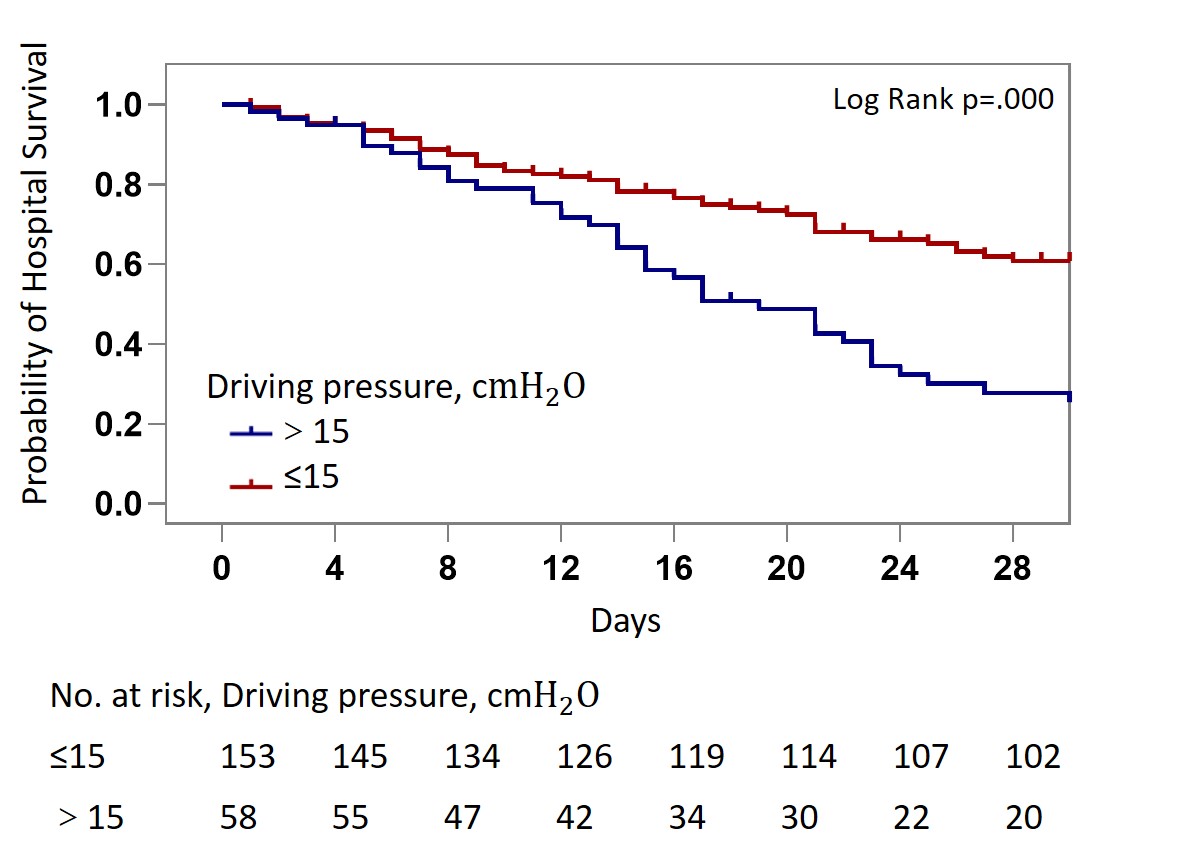

Supplement: Supplementary file 1 — Additional file 1: eFig 1. Probability of hospital survival by driving pressure. Patients with a driving pressure greater than 15 cmH2O on day 1 of mechanical ventilation after ARDS diagnosis had a higher mortality. [file 13054_2020_3112_MOESM1_ESM.jpg]

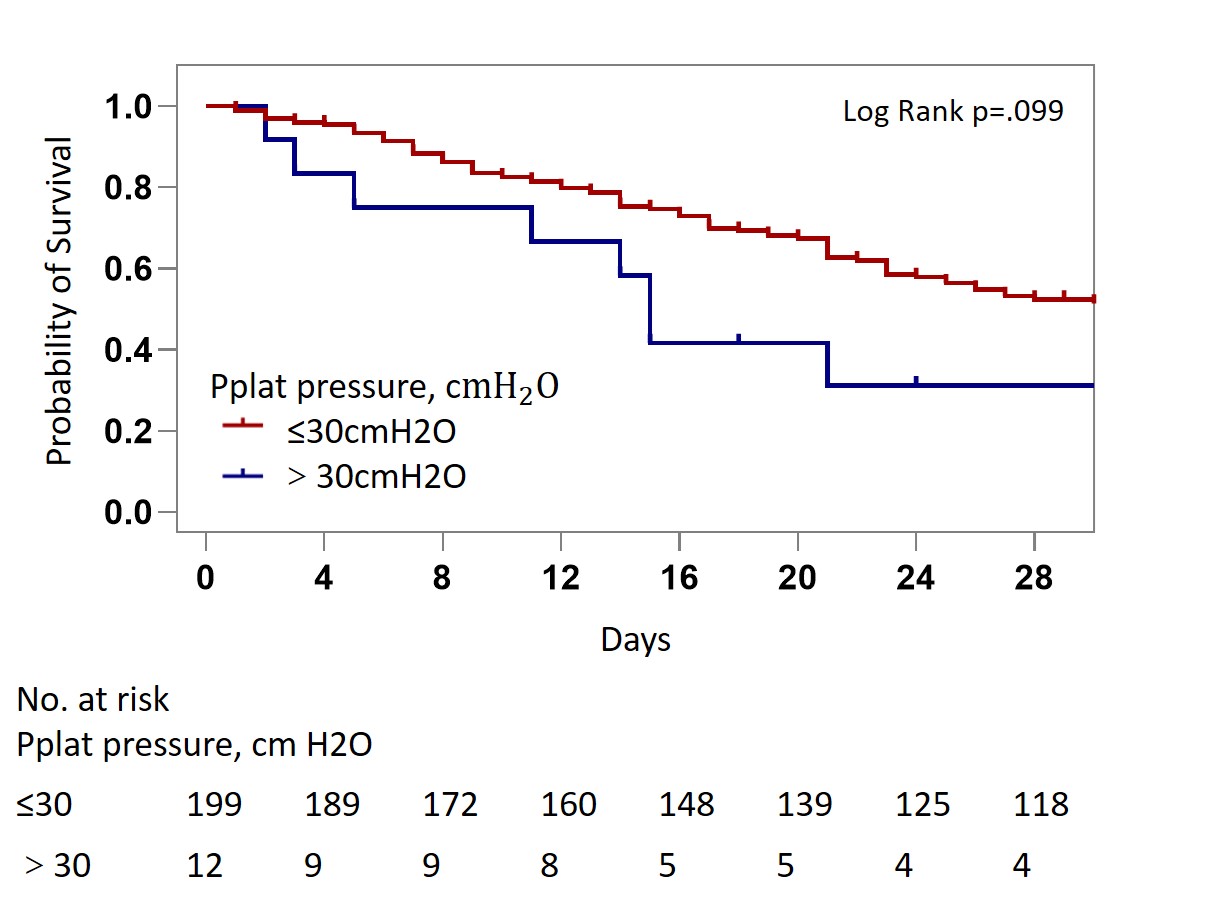

Supplement: Supplementary file 2 — Additional file 2: eFig 2. Probability of hospital survival by plateau pressure. Patients with a plateau pressure of greater than 30 cmH2O on day 1 of mechanical ventilation after ARDS diagnosis had mortality similar to that of patients with a plateau pressure of less than 30 cmH2O. [file 13054_2020_3112_MOESM2_ESM.jpg]

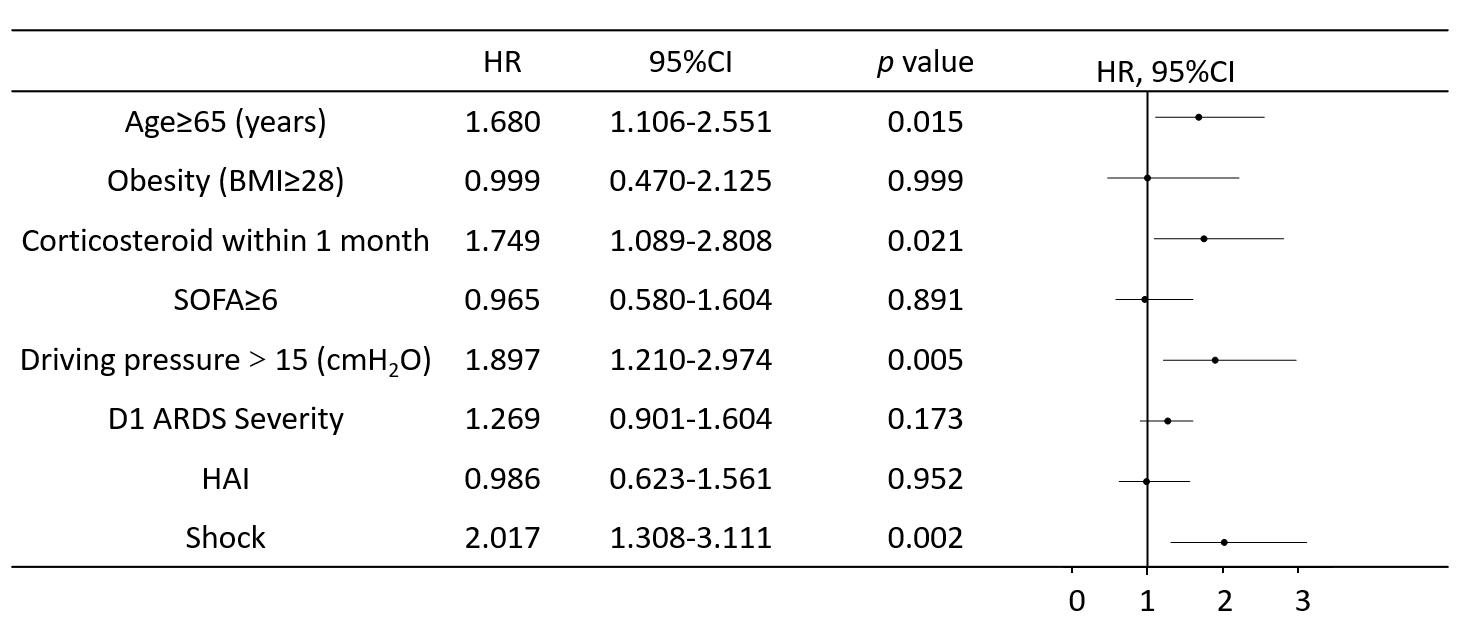

Supplement: Supplementary file 3 — Additional file 3: eFig 3. Logistic regression of hospital mortality. [file 13054_2020_3112_MOESM3_ESM.jpg]

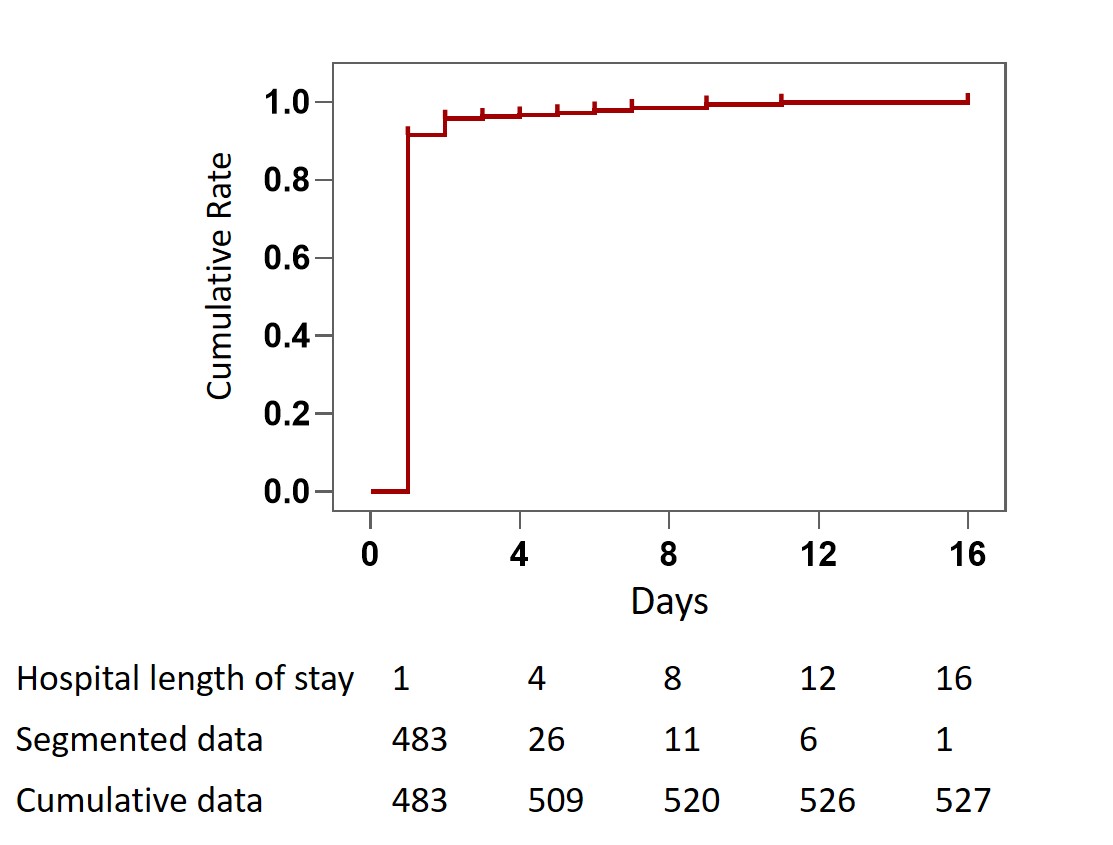

Supplement: Supplementary file 4 — Additional file 4: eFig 4. Time-to-event analysis of the time course of ARDS onset. [file 13054_2020_3112_MOESM4_ESM.jpg]
