# Supplementary material for: Incidence and outcomes of acute respiratory distress syndrome in intensive care units of mainland China: a multicentre prospective longitudinal study
Source: Crit Care. 2020 Aug 20;24:515. doi: 10.1186/s13054-020-03112-0 (PMC7439799; doi:10.1186/s13054-020-03112-0)
Supplement: Supplementary file 5 — Additional file 5. Characteristics of chinese hospitals and ICUs including process of care and ICU delivery systems. [file 13054_2020_3112_MOESM5_ESM.docx]

Characteristics of Chinese hospitals and ICUs including process of care and ICU delivery systems

All participating centers were closed ICUs in tertiary teaching hospitals in metropolitan cities, managed by full-time ICU doctors.

- The admission criteria is as follows:

1. Recoverable acute, life threatening organ dysfunction or failure;
2. The presence of potential life risks who may be at reduced risk of death through intensive monitoring and effective treatment
3. Chronic comorbid conditions with life-threatening acute severe illness, who may return to their original state after intensive care.

Exclusion criteria

Patients with end-state chronic disease, irreversible disease, and inability to benefit from intensive care are generally not admitted into ICU.

- Discharge criteria

1. Physical status of the patients has been stabilized and no longer need ICU intensive care. Usually the stabilization of organ failure.

2. Patients who withdraw life sustaining support.

- ICU triage

The aim of triage is to save as many lives as possible.

1. Patients should be admitted if they could benefit from with decreased risk of death.
2. Patients with reversible medical conditions
3. Wishes of patients or surrogate.
4. Anticipated quality of life.
5. Little likelihood of survival patients should not be admitted to ICU.
6. Age.
7. Likely duration of ICU stay.
8. Number of ICU beds/staff.

- Referral patterns

1. The clinicians think the condition of the patients who were treated in general wards or emergency room meet the admission criteria of ICU, they would consult the doctor in ICU. If the intensivists determined that the patients meet the criteria for admission to ICU，the patients would be transferred to ICU for intensive care.
2. If the intensivists determined that the patients meet the criteria for transferring to general ward, they would consult chief resident of general wards and decide the transfer of the patients.
3. If a patient's condition in the community hospital gets worse and exceeds the hospital's treatment capacity or the treatment effect is not satisfactory, the hospital would apply for the consultation of ICU physicians in the tertiary hospitals, if the ICU physicians in the tertiary hospitals confirm the condition and agree to transfer the patient, the patient would be transferred to the tertiary hospitals.

- Role of respiratory therapists and critical care nurses

1. Role of respiratory therapists
   1. Monitoring and evaluation of respiratory therapy.
   2. Respiratory physiological monitoring
   3. Mechanical ventilation, including self-examination and debugging before the use of ventilator, regulation of mode and parameters, ventilator-related complications such as ventilator-related lung injury, prevention and treatment of ventilator-related pneumonia, and mechanical ventilation evacuation.
   4. Assist doctors in the establishment of artificial airway, including intubation through nasal and breath tubes and tracheotomy, which can be independently operated in emergency situations.
   5. Artificial airway management, including catheter position management, artificial airway balloon management, artificial airway temperature, humidification, artificial airway removal, etc.
   6. Oxygen therapy, including oxygen concentration regulation, oxygen therapy device selection and use, oxygen therapy effect evaluation.
   7. Nebulization therapy, including the choice of nebulized drugs and devices, the use of devices, the monitoring of process, and the evaluation of effect.
   8. Chest physical therapy, including postural drainage, chest vibration sputum excretion, guiding cough, sputum aspiration through nose, mouth and artificial airway negative pressure, lung dilation treatment, etc.
   9. Respiratory rehabilitation exercises, including controlled breathing techniques, breathing muscle training, etc.
   10. Central vein catheterization and arterial catheterization.
   11. Tracheoscopy.
   12. Examination of lung function.
   13. Collection of sputum specimens.
   14. Gas metabolism analysis.
   15. Arterial blood gas analysis.
   16. In-hospital/out-patient transport of mechanically ventilated patients.
   17. Participated in cardiopulmonary resuscitation, and mastered the use of simple breathing apparatus and cardiac electrical defibrillator.
   18. Hyperbaric oxygen chamber treatment.
   19. Sleep apnea monitoring.
   20. Management of respiratory treatment-related instruments, including the cleaning, disinfection and performance testing of the ventilator; Cleaning, disinfection and installation of ventilator pipeline; Maintenance of atomizing device, heating and humidifying device, tracheal mirror, vibration sputum extractor, negative pressure sputum aspirator, heart electric defibrillator, blood gas analyzer.
   21. For family therapy, RT shall guide patients and their families in the use and maintenance of ventilator and related respiratory treatment instruments, ensure their safe and effective use, and guide patients in atomization inhalation therapy, respiratory rehabilitation exercise, etc.
   22. Smoking cessation guidance.
   23. Health education.

- Role of critical care nurses

1. Intensive observation and evaluation of the patient's condition, preparing for emergency medicine and equipment and complete nursing record；
2. Correctly follow the doctor's prescription, and master nursing techniques and operating procedures of instruments and equipment.
3. Be ready to cooperate with the doctors to rescue the patients;
4. Strictly implement the disinfection and isolation system to prevent and control the occurrence of hospital infection；
5. Carry out health education for the patients；
6. Ensure the safety of patients, implement policies and measures to prevent falls, pressure sores and pipeline slippage, and prevent related complications.
