# Supplementary material for: Incidence and outcomes of acute respiratory distress syndrome in intensive care units of mainland China: a multicentre prospective longitudinal study
Source: Crit Care. 2020 Aug 20;24:515. doi: 10.1186/s13054-020-03112-0 (PMC7439799; doi:10.1186/s13054-020-03112-0)
Supplement: Supplementary file 6 — Additional file 6: eTable 1. Characteristics of participating centers. [file 13054_2020_3112_MOESM6_ESM.docx]

eTable 1 Characteristics of participating centers

| Characteristics | Participating centers  (n=17) |
| --- | --- |
| Type of hospital, n (%) |  |
| University affiliated | 11（64.7） |
| Public | 6（35.3） |
| Type of ICU, n (%) |  |
| Respiratory /Medical | 16（88.9） |
| General | 2（11.1） |
| Number of ICU beds  Median [IQR] | 20（15-28） |
| Accounting for all hospital beds (%) | 1.2（0.8-1.8） |
| staff available in ICU，n（%） |  |
| Doctorsa (IQR):ICU beds | 0.7 (0.6-0.9):1 |
| Nurses (IQR):ICU beds | 1.9 (1.5-2.4):1 |
| Respiratory therapists (IQR):ICU beds | 0.11 (0.08-0.16):1 |
| Technology available in ICU, n (%) |  |
| Noninvasive mechanical ventilation | (100%) |
| Invasive mechanical ventilation | (100%) |
| ECMO | 3（17.6） |
| HFOV | 1（0.6） |
| Inhaled nitric oxide | 0（0） |

a, include physicians, residents and fellows. ICU, intensive care unit; IQR, interquartile range; ECMO, extracorporeal membrane oxygenation; HFOV, high frequency oscillatory ventilation.
