# Supplementary material for: Incidence and outcomes of acute respiratory distress syndrome in intensive care units of mainland China: a multicentre prospective longitudinal study
Source: Crit Care. 2020 Aug 20;24:515. doi: 10.1186/s13054-020-03112-0 (PMC7439799; doi:10.1186/s13054-020-03112-0)
Supplement: Supplementary file 7 — Additional file 7: eTable 2. Resources and types of medical expenses of acute respiratory distress syndrome patients. [file 13054_2020_3112_MOESM7_ESM.docx]

eTable 2 Resources and types of medical expenses of acute respiratory distress syndrome patients

| Parameter | **ARDS**  **n=527** | **Mild**  **n=51** | **Moderate**  **n=250** | **Severe**  **n=226** |
| --- | --- | --- | --- | --- |
| Resources |  |  |  |  |
| Emergency room | 179 (34.0) | 24 (47.1) | 84 (33.7) | 71 (31.4) |
| Other wards | 149 (28.3) | 12 (23.5) | 75 (30.1) | 62 (27.4) |
| Other ICU | 44 (8.4) | 4 (7.8) | 17 (6.8) | 23 (10.2) |
| Other hospital | 21 (4.0) | 2 (3.9) | 13 (5.2) | 6 (2.7) |
| Emergency surgery | 6 (1.1) | 0 (0.0) | 3 (1.2) | 3 (1.3) |
| Selected surgery | 127 (24.1) | 9 (17.6) | 57 (22.9) | 61 (27.0) |
| Medical expenses |  |  |  |  |
| Medical insurance | 278 (53.0) | 27 (52.9) | 130 (52.2) | 121 (53.0) |
| Free medical care | 16 (3.0) | 1 (2.0) | 6 (2.4) | 9 (4.0) |
| Commercial insurance | 3 (0.6) | 0 (0.0) | 2 (0.8) | 1 (0.4) |
| Rural cooperative medical care | 82 (15.6) | 5 (9.8) | 37 (14.9) | 40 (17.8) |
| Self-paying medical care | 102 (19.4) | 11 (21.6) | 55 (22.1) | 36 (16.0) |
| Other medical care | 44 (8.4) | 7 (13.7) | 19 (7.6) | 18 (8.0) |
