# Supplementary material for: Incidence and outcomes of acute respiratory distress syndrome in intensive care units of mainland China: a multicentre prospective longitudinal study
Source: Crit Care. 2020 Aug 20;24:515. doi: 10.1186/s13054-020-03112-0 (PMC7439799; doi:10.1186/s13054-020-03112-0)
Supplement: Supplementary file 8 — Additional file 8: eTable 3. Laboratory findings of acute respiratory distress syndrome patients. [file 13054_2020_3112_MOESM8_ESM.docx]

eTable 3 Laboratory findings in acute respiratory distress syndrome patients

| Laboratory findings | **ARDS**  **n=527** | **Mild**  **n=51** | **Moderate**  **n=250** | **Severe**  **n=226** | ***P***  **value** |
| --- | --- | --- | --- | --- | --- |
| D1  Blood routine |  |  |  |  |  |
| WBC, *10^9^/L | 10.7 (7.0, 15.6) | 11.0 (6.7, 17.0) | 10.6 (7.2, 14.9) | 10.7 (6.5, 15.9) | 0.882 |
| Lymphocyte, *10^9^/L | 6.4 (3.7, 11.0) | 5.8 (3.8, 12.5) | 6.6 (3.9, 11.6) | 6.1 (3.6, 10.5) | 0.453 |
| Hemoglobin, g/L | 114.0 (96.0, 132.0) | 104.5 (95.8, 129.2) | 113.0 (94.0, 130.2) | 116.0 (97.0, 135.0) | 0.331 |
| Platelet, *10^9^/L | 158.0 (106.0, 232.0) | 165.5 (97.0, 235.0) | 162.0 (108.2, 230.0) | 153.0 (108.0, 236.0) | 0.866 |
| D2  Blood routine |  |  |  |  |  |
| WBC, *10^9^/L | 10.3 (7.3, 15.2) | 10.7 (7.3, 14.8) | 9.9 (6.8, 14.5) | 10.7 (7.5, 16.1) | 0.242 |
| Lymphocyte, *10^9^/L | 6.2 (3.7, 10.5) | 6.9 (3.8, 13.2) | 6.2 (3.9, 11.3) | 5.7 (3.5, 9.4) | 0.092 |
| Hemoglobin, g/L | 106.0 (89.0, 124.0) | 98.0 (89.0, 122.0) | 106.0 (84.0, 123.0) | 109.5 (92.0, 126.8) | 0.149 |
| Platelet, *10^9^/L | 150.0 (95.0, 221.0) | 155.0 (92.0, 228.0) | 154.0 (91.0, 228.0) | 144.0 (99.0, 199.5) | 0.502 |
| D1 |  |  |  |  |  |
| ALT, IU/L | 33.0 (19.0, 61.4) | 29.0 (19.0, 58.0) | 33.0 (18.0, 70.0) | 35.0 (23.0, 60.0) | 0.332 |
| AST, IU/L | 48.0 (29.0, 94.4) | 35.0 (23.0, 75.5) | 45.0 (26.0, 83.0) | 54.6 (34.6, 105.5) | 0.005 |
| Total bilirubin, umol/L | 12.8 (8.2, 21.8) | 14.5 (8.3, 24.7) | 13.0 (8.5, 22.9) | 12.5 (7.9, 20.8) | 0.424 |
| Direct bilirubin, umol/L | 5.9 (3.6, 10.2) | 6.4 (3.9, 15.7) | 6.0 (3.7, 11.0) | 5.7 (3.4, 9.2) | 0.443 |
| Albumin, g/L | 29.0 (24.7, 32.8) | 30.5 (27.4, 33.2) | 28.7 (24.3, 32.0) | 29.0 (24.4, 33.2) | 0.287 |
| blood urine nitrogen, mmol/L | 7.5 (5.3, 11.7) | 7.0 (3.8, 16.5) | 7.0 (5.3, 10.0) | 8.0 (5.5, 12.9) | 0.062 |
| Creatinine, umol/L | 75.7 (55.2, 109.7) | 64.5 (47.0, 145.4) | 73.4 (53.5, 108.8) | 77.8 (57.8, 105.9) | 0.342 |
| Glucose, mmol/L | 8.1 (6.3, 11.7) | 7.7 (5.7, 13.4) | 8.3 (6.1, 11.8) | 8.2 (6.8, 11.5) | 0.633 |
| K, mmol/L | 3.9 (3.5, 4.3) | 4.0 (3.6, 4.6) | 4.0 (3.6, 4.4) | 3.8 (3.4, 4.2) | 0.218 |
| Na, mmol/L | 137.8 (133.9, 141.0) | 138.3 (133.3, 141.8) | 137.0 (133.0, 141.0) | 138.0 (134.2, 141.7) | 0.543 |
| Cl, mmol/L | 102.5 (99.0, 107.0) | 104.3 (99.3, 108.5) | 102.0 (99.0, 107.0) | 102.0 (98.6, 107.0) | 0.534 |
| TNI, ng/ml | 0.036 (0.012, 0.087) | 0.019 (0.010, 0.063) | 0.034 (0.012, 0.072) | 0.045 (0.015, 0.160) | 0.041 |
| BNP, pg/ml | 153.0 (47.4, 510.4) | 117.1 (42.6, 492.7) | 154.6 (60.4, 581.0) | 153.5 (46.0, 495.5) | 0.839 |
| NT pro-BNP, pg/ml | 786.6 (230.8, 2914.0) | 643.5 (138.0, 2771.0) | 926.0 (285.0, 2936.0) | 751.0 (199.8, 2914.0) | 0.822 |
| PCT, mg/ml | 1.3 (0.4, 6.6) | 2.5 (0.3, 18.5) | 1.1 (0.4, 8.0) | 1.4 (0.5, 5.2) | 0.460 |
| CRP, mg/L | 113.9 (49.2, 198.0) | 120.1 (43.8, 200.0) | 104.8 (46.3, 198.0) | 129.9 (53.9, 197.2) | 0.621 |
| PT, s | 14.3 (12.6, 16.0) | 14.1 (12.2, 16.5) | 14.5 (12.6, 16.2) | 14.3 (12.7, 15.7) | 0.857 |
| Fib, g/L | 4.9 (3.5, 6.4) | 5.5 (3.8, 7.1) | 5.0 (3.5, 6.6) | 4.8 (3.4, 6.3) | 0.146 |
| D-Dimer, mg/L | 3.3 (1.4, 10.7) | 2.9 (1.1, 8.0) | 2.9 (1.3, 9.1) | 3.8 (1.6, 15.4) | 0.165 |
| D2 |  |  |  |  |  |
| ALT, IU/L | 29.8 (19.0, 65.8) | 34.0 (21.0, 89.0) | 28.5 (16.5, 65.0) | 30.9 (20.3, 68.4) | 0.404 |
| AST, IU/L | 45.2 (28.0, 95.1) | 47.9 (27.0, 105.0) | 44.0 (25.0, 85.5) | 45.5 (31.4, 107.3) | 0.237 |
| Total bilirubin, umol/L | 13.2 (7.5, 21.2) | 13.2 (6.3, 22.0) | 13.4 (8.0, 21.9) | 13.1 (7.5, 20.6) | 0.715 |
| Direct bilirubin, umol/L | 6.4 (3.7, 12.0) | 5.9 (3.0, 13.9) | 6.6 (3.8, 11.9) | 6.2 (3.8, 12.3) | 0.903 |
| Albumin, g/L | 28.0 (24.4, 31.4) | 28.9 (25.9, 34.6) | 27.6 (25.0, 31.0) | 28.0 (24.0, 31.4) | 0.321 |
| blood urine nitrogen, mmol/L | 8.2 (5.6, 13.4) | 7.7 (4.2, 14.7) | 7.6 (5.2, 12.0) | 9.3 (6.2, 14.3) | 0.060 |
| Creatinine, umol/L | 69.1 (54.4, 119.3) | 56.7 (45.2, 151.0) | 68.1 (55.5, 117.8) | 73.0 (55.8, 117.8) | 0.408 |
| Glucose, mmol/L | 8.3 (6.5, 11.2) | 7.7 (6.0, 9.9) | 8.0 (6.5, 10.9) | 8.6 (6.5, 11.7) | 0.098 |
| K, mmol/L | 4.0 (3.7, 4.4) | 3.9 (3.7, 4.3) | 3.9 (3.6, 4.3) | 4.1 (3.7, 4.4) | 0.013 |
| Na, mmol/L | 139.0 (136.0, 143.0) | 139.0 (137.4, 143.0) | 138.3 (135.0, 142.0) | 139.2 (136.1, 143.0) | 0.275 |
| Cl, mmol/L | 104.0 (100.0, 108.0) | 106.0 (100.3, 109.0) | 104.0 (100.0, 108.0) | 103.8 (100.0, 107.2) | 0.713 |
| TNI,ng/ml | 0.050 (0.017, 0.150) | 0.047 (0.010, 0.132) | 0.042 (0.014, 0.080) | 0.055 (0.025, 0.364) | 0.009 |
| BNP, pg/ml | 157.0 (57.0, 703.0) | 198.0 (55.5, 397.0) | 215.8 (66.6, 905.2) | 140.0 (50.0, 703.0) | 0.797 |
| NT pro-BNP, pg/ml | 1189.0 (343.3, 3718.0) | 446.0 (156.9, 3567.0) | 980.3 (355.5, 2912.0) | 1857.0 (436.8, 4110.0) | 0.176 |
| PCT, mg/ml | 1.9 (0.5, 10.0) | 1.7 (0.3, 22.8) | 1.5 (0.5, 8.1) | 2.6 (0.6, 12.0) | 0.358 |
| CRP, mg/L | 129.0 (62.1, 200.0) | 159.0 (39.6, 196.4) | 129.0 (71.0, 200.0) | 125.0 (59.8, 200.0) | 0.862 |
| PT, s | 14.5 (12.8, 16.2) | 14.1 (12.8, 15.7) | 14.8 (12.9, 16.5) | 14.2 (12.7, 15.9) | 0.158 |
| Fib, g/L | 4.8 (3.4, 6.3) | 5.3 (3.7, 7.7) | 4.9 (3.4, 6.6) | 4.5 (3.3, 6.1) | 0.094 |
| D-Dimer, mg/L | 4.8 (2.3, 11.4) | 2.8 (1.0, 10.1) | 4.2 (2.2, 8.8) | 6.2 (2.8, 19.4) | 0.025 |

WBC: white blood cell; ALT: alanine aminotransferase; AST: aspartate aminotransferase；K：potassium; Na:sodium; Cl:chloride; TNI: troponin I; BNP: Brain natriuretic peptide; NT pro-BNP: N-terminal pro-B-type natriuretic peptide; PCT: procalcitonin; CRP: C reactive protein; PT: prothrombin time；Fib: fibrinogen
