# Supplementary material for: Incidence and outcomes of acute respiratory distress syndrome in intensive care units of mainland China: a multicentre prospective longitudinal study
Source: Crit Care. 2020 Aug 20;24:515. doi: 10.1186/s13054-020-03112-0 (PMC7439799; doi:10.1186/s13054-020-03112-0)
Supplement: Supplementary file 9 — Additional file 9: eTable 4. Characteristics of ARDS patients treated with noninvasive ventilation by severity category at diagnosis. [file 13054_2020_3112_MOESM9_ESM.docx]

eTable 4. Characteristics of ARDS Patients Treated With Noninvasive Ventilation by Severity Category at Diagnosis

| Parameter | **ARDS**  **n=527** | **Mild**  **n=51** | **Moderate**  **n=250** | **Severe**  **n=226** | ***P***^a^  **Value** |
| --- | --- | --- | --- | --- | --- |
| D1 NPPV^b^ | 143 (27.1) | 13 (25.5) | 83 (33.2) | 47 (20.8) | 0.002 |
| D1 NPPV mode |  |  |  |  |  |
| BiPAP | 114 (83.8) | 11 (91.7) | 67 (82.7) | 36 (83.7) |  |
| CPAP | 22 (16.2) | 1 (8.3) | 14 (17.3) | 7 (16.3) |  |
| D1 IPAP, median (IQR), cmH_2_O | 13.5 (12.0-15.0) | 12.0 (12.0-14.0) | 12.5 (10.5-15.0) | 14.0 (12.0-15.0) | 0.574 |
| D1 EPAP, median (IQR), cmH_2_O | 5.0 (5.0-7.0) | 5.0 (5.0-6.0) | 6.0 (5.0-7.0) | 6.0 (5.0-8.0) | 0.264 |
| NPPV during ICU^c^ | 200 (38.0) | 16 (31.4) | 117 (46.8) | 67 (29.6) | 0.000 |
| NPPV complications |  |  |  |  |  |
| NPPV barotrauma | 22 (4.2) | 0 (0.0) | 10 (4.0) | 12 (5.3) | 0.089 |
| Subcutaneous emphysema | 6 (1.1) | 0 (0.0) | 1 (0.4) | 5 (2.2) |  |
| Mediastinal emphysema | 6 (1.1) | 0 (0.0) | 4 (1.6) | 4 (1.8) |  |
| Pneumothorax | 10 (1.9) | 0 (0.0) | 5 (2.0) | 5 (2.2) |  |
| Interstitial emphysema | 1 (0.2) | 0 (0.0) | 1 (0.4) | 0 (0.0) |  |
| Facial pressure sore | 2 (0.4) | 0 (0.0) | 2 (0.8) | 0 (0.0) |  |
| Distention | 5 (0.9) | 0 (0.0) | 1 (0.4) | 4 (1.8) |  |

NPPV: non-invasive positive pressure ventilation; BiPAP: Bilevel positive airway pressure CPAP: continuous positive airway pressure; IPAP: inspiratory positive airway pressure ; EPAP: expiratory positive airway pressure; ICU: intensive care unit

^a^ P value represents comparisons across the ARDS severity categories for each variable.

^b^ We had excluded the patients who used HFNC (high flow nasal cannulae) after confirming ARDS by NPPV when analyzing the data of NPPV patients.

^c^ The numbers of patients who used NPPV during ICU was the sum of three parts: the patients who initially used NPPV for treatment, the patients who used NPPV only for comfirming ARDS and the patients who weaned from IPPV.
