# Supplementary material for: Incidence and outcomes of acute respiratory distress syndrome in intensive care units of mainland China: a multicentre prospective longitudinal study
Source: Crit Care. 2020 Aug 20;24:515. doi: 10.1186/s13054-020-03112-0 (PMC7439799; doi:10.1186/s13054-020-03112-0)
Supplement: Supplementary file 10 — Additional file 10: eTable 5. Fluid balance of acute respiratory distress syndrome patients. [file 13054_2020_3112_MOESM10_ESM.docx]

eTable 5 Fluid balance of acute respiratory distress syndrome patients

| Parameter | **ARDS**  **n=527** | **Mild**  **n=51** | **Moderate**  **n=250** | **Severe**  **n=226** | ***P^a^***  **value** |
| --- | --- | --- | --- | --- | --- |
| D1 fluid balance | 379 (-123, 1142) | 227 (-320, 812) | 227 (-196, 1022) | 400 (-5, 1190) | 0.037 |
| Accumulative fluid balance D1-D2 | 850 (-160, 1915) | 523 (-516, 2153) | 744 (-165, 1738) | 1014 (109, 2141) | 0.081 |
| Accumulative fluid balance D1-D3 | 1077 (-398, 2806) | 566 (-427, 2875) | 857 (407, 2432) | 1404 (-371, 2990) | 0.075 |
| Accumulative fluid balance D1-D4 | 1212 (-488, 3192) | 281 (-898, 2625) | 1117 (-500, 3090) | 1596 (-324, 3579) | 0.074 |
| Accumulative fluid balance D1-D5 | 1391 (-587, 3590) | 179 (-1409, 3340) | 1154 (-572, 3530) | 1614 (-376, 3616) | 0.195 |
| Accumulative fluid balance D1-D6 | 1424 (-743, 3899) | 191 (-1358, 3213) | 1239 (-848, 4008) | 2022 (-498, 3769) | 0.265 |
| Accumulative fluid balance D1-D7 | 1669 (-1101, 4351) | 132 (-1188, 3796) | 1525 (-1093, 4403) | 2302 (-526, 4303) | 0.366 |
| Urine output D1 | 1200 (650,1986) | 1130 (650, 2125) | 1200 (650, 2000) | 1200 (688, 1955) | 0.943 |
| Accumulative Urine output D1-D2 | 3172 (1992, 4550) | 3500 (2360, 4520) | 3150 (1884, 4520) | 3160 (1942, 4417) | 0.634 |
| Accumulative Urine output D1-D3 | 5370 (3422, 7350) | 5575 (3545, 7480) | 5370 (3430, 7150) | 5252 (3280, 7350) | 0.711 |
| Accumulative Urine output D1-D4 | 7712 (4621, 10297) | 8050 (5500, 10470) | 7620 (4740, 10300) | 7732 (4467, 10170) | 0.713 |
| Accumulative Urine output D1-D5 | 9987 (5982, 12937) | 10330 (7125, 13060) | 10200 (5840, 13060) | 9905 (5787, 12687) | 0.899 |
| Accumulative Urine output D1-D6 | 12300 (7218, 15657) | 12350 (7500, 14610) | 12187 (7330, 15840) | 12342 (6987, 15840) | 0.965 |
| Accumulative Urine output D1-D7 | 13960 (7941, 19056) | 12555 (7620, 17115) | 13695 (8260, 17850) | 14450 (7700, 18503) | 0.641 |
| Colloid input on D1 | 0 (0, 300) | 0 (0, 100) | 0 (0, 200) | 50 (0, 400) | 0.022 |
| Accumulative colloid input D1-D2 | 200 (0, 650) | 100 (0, 630) | 200 (0, 500) | 200 (0, 800) | 0.234 |
| Accumulative colloid input D1-D3 | 300 (0, 1025) | 250 (0, 810) | 300 (0, 025) | 400 (0, 1200) | 0.298 |
| Accumulative colloid input D1-D4 | 550 (0, 1350) | 300 (0, 897) | 500 (0, 1262) | 600 (25, 1500) | 0.080 |
| Accumulative colloid input D1-D5 | 750 (75, 1700) | 325 (0, 987) | 700 (50, 1702) | 800 (125, 1780) | 0.153 |
| Accumulative colloid input D1-D6 | 800 (100, 1940) | 525 (0, 1375) | 800 (100, 1962) | 950 (200, 1925) | 0.306 |
| Accumulative colloid input D1-D7 | 1000 (200, 2135) | 600 (0, 1450) | 900 (200, 2102) | 1090 (225, 2300) | 0.493 |

a *P* value represents comparisons across the ARDS severity categories for each variable.

D1: the day of ARDS diagnosis; D2: the second day of ARDS diagnosis, and so on.
