# Supplementary material for: Incidence and outcomes of acute respiratory distress syndrome in intensive care units of mainland China: a multicentre prospective longitudinal study
Source: Crit Care. 2020 Aug 20;24:515. doi: 10.1186/s13054-020-03112-0 (PMC7439799; doi:10.1186/s13054-020-03112-0)
Supplement: Supplementary file 11 — Additional file 11: eTable 6. Comparison of survivors versus non-survivors in patients with ARDS. [file 13054_2020_3112_MOESM11_ESM.docx]

eTable 6. Comparison of survivors versus non-survivors in patients with ARDS

| Parameter | **Survival**  **(n=283)** | **Non-Survival**  **(n=244)** | ***P***  **Value** | **HR** | **95%CI** |
| --- | --- | --- | --- | --- | --- |
| APACHEⅡ | 15.2±7.2 | 19.6±8.0 | 0.000 | 1.078 | 1.052-1.105 |
| SOFA | 6.4±3.6 | 8.4±3.9 | 0.000 | 1.153 | 1.096-1.213 |
| Age, mean, y | 52.2±16.9 | 58.8±17.4 | 0.000 | 1.022 | 1.011-1.033 |
| Men, No(%) | 187 (66.1) | 182(74.6) | 0.033 | 1.507 | 1.032-2.201 |
| Obesity, No(%) | 45 (16.3) | 20 (8.3) | 0.006 | 0.462 | 0.265-0.808 |
| Corticosteroid or immunosuppressive within 1month | 41 (14.9) | 69 (28.3) | 0.000 | 2.250 | 1.459-3.471 |
| D1 NPPV | 82 (29.0) | 61 (25.0) |  |  |  |
| NPPV during ICU | 118 (41.7) | 82 (33.6) | 0.056 | 0.708 | 0.496-1.010 |
| D1 IPPV | 150 (53.0) | 149 (61.1) |  |  |  |
| IPPV during ICU | 178 (62.9) | 222 (91.0) | 0.000 | 2.931 | 1.872-4.590 |
| PEEP, Median (IQR), cmH_2_O | 8 (6-12) | 9 (6-12) | 0.646 | 1.003 | 0.952-1.057 |
| V_T_, Median (IQR), ml/kg ideal weight | 6.9 (6.0-7.7) | 6.8 (5.9-8.0) | 0.919 | 1.004 | 0.901-1.117 |
| Pplat^a^, Median (IQR), cmH_2_O | 20 (15-23) | 23 (18-28) | 0.000 | 1.081 | 1.033-1.131 |
| Driving pressure^a^, Median (IQR), cmH_2_O | 11 (7-14) | 20 (15-23) | 0.000 | 1.104 | 1.049-1.162 |
| D1 PaO2/FIO2 , Median (IQR), mmHg | 127 (87-170) | 100 (70-135) | 0.000 | 0.993 | 0.990-0.996 |
| NMBAs | 43 (15.2) | 68 (27.9) | 0.000 | 2.156 | 1.405-3.310 |
| RM | 16 (5.7) | 28 (11.5) | 0.016 | 2.163 | 1.141-4.102 |
| PPV | 34 (12.0) | 51 (20.9) | 0.006 | 1.935 | 1.206-3.105 |
| ECMO | 21 (7.4) | 40 (16.4) | 0.001 | 2.446 | 1.399-4.278 |
| HFOV | 0 (0.0) | 3 (1.2) | 0.061 |  |  |
| High dose corticosteroid^c^ | 79 (27.9) | 72 (29.5) | 0.687 | 1.081 | 0.740-1.578 |
| Non-respiratory organ failure | 61 (21.6) | 180 (73.9) | 0.000 | 10.236 | 6.847-15.301 |
| Shock | 37 (13.1) | 154 (53.4) | 0.000 | 11.504 | 7.461-17.740 |
| ICU length of stay, d | 13 (8,26) | 10 (5,17) | 0.000 |  |  |
| Hospital length of stay, d | 23 (15,35) | 12 (7,21) | 0.000 |  |  |

ARDS: acute respiratory distress syndrome; NPPV: non-invasive positive pressure ventilation; IPPV: invasive positive pressure ventilation; ICU: intensive care unit；PEEP: positive end-expiratory pressure; V_T_: tidal volume; Pplat : plateau pressure; organ failure: SOFA scores ≥3 referring to one organ were defined as failure of that organ; NMBAs: Neuromuscular blockade; RM: lung recruitment manoeuvre; PEEP: positive end expiratory pressure; PPV prone position ventilation; EMCO: extracorporeal membrane oxygenation; HFOV :high-frequency oscillary ventilation

a Plateau pressure values, driving pressure values, airway resistance values and respiratory compliance values are limited to patients in whom this value was reported, the number of measured patients is 211 cases, Patients receiving HFOV or ECMO were also excluded.

bAll the patients who withdrawed and discharged from the hospital were confirmed dead on the day of withdrawal, therefore when ICU and hospital mortality were calculated, the withdrawed patients were included

^c^ High-dose corticosteroids was defined as doses that were equal to or greater than the equivalent of 1 mg/kg of prednisolone.
